# PSoC-Stat: A single chip open source potentiostat based on a Programmable System on a Chip

**DOI:** 10.1371/journal.pone.0201353

**Published:** 2018-07-25

**Authors:** Prattana Lopin, Kyle V. Lopin

**Affiliations:** 1 Department of Biology, Faculty of Science, Naresuan University, Phitsanulok, Thailand; 2 Department of Physics, Faculty of Science, Naresuan University, Phitsanulok, Thailand; University of California Santa Cruz, UNITED STATES

## Abstract

In this paper we demonstrate a potentiostat built with a single commercially available integrated circuit (IC) that does not require any external electronic components to perform electrochemical experiments. This is done using the capabilities of the Programmable System on a Chip (PSoC^®^) by Cypress Semiconductor, which integrates all of the necessary electrical components. This is in contrast to other recent papers that have developed potentiostats but require technical skills or specialized equipment to produce. This eliminates the process of having to make a printed circuit board and soldering on electronic components. To control the device, a graphical user interface (GUI) was developed in the python programming language. Python is open source, with a style that makes it easy to read and write programs, making it an ideal choice for open source projects. As the developed device is open source and based on a PSoC, modification to implement other electrochemical techniques is straightforward and only requires modest programming skills, but no expensive equipment or difficult techniques. The potentiostat developed here adds to the growing amount of open source laboratory equipment. To demonstrate the PSoC potentiostat in a wide range of applications, we performed cyclic voltammetry (to measure vitamin C concentration in orange juice), amperometry (to measure glucose with a glucose strip), and stripping voltammetry experiments (to measure lead in water). The device was able to perform all experiments and could accurately measure Vitamin C, glucose, and lead.

## Introduction

Electrochemistry studies the movement of electrons during chemical reactions and is important for numerous fields including for chemistry (analytical chemistry) [1], biology (neurotransmitter release) [2], material science (electrodeposition, anodization) [3], energy storage (batteries) [4], medicine (glucose sensors) [5], and environmental sensing (heavy metal detection) [6]. A chemical reaction with a transfer of charge between molecules is called an oxidation-reduction reaction. An electrochemical reaction occurs when there is a chemical reaction and a transfer of charge to an external source [7]. By measuring the amount of charge moving through the external source, the chemical reaction rate can be determined. This allows for the monitoring of chemical concentrations and reactions by external electronics [8].

The most common electrochemical device used is the battery, where internal movement of ions between electrodes, releases electrons that can be used to do electrical work. Electrochemistry is also widely used in analytical chemistry to measure concentrations and reaction rates. The glucose meter found in most drug stores uses electrochemistry to measure blood glucose levels. As glucose is not directly electrochemically active, an enzyme is used to catalyze the oxidation of glucose. First generation glucose biosensors use glucose oxidase (GOx) to oxidize glucose to gluconic acid and hydrogen peroxide [9]. The produced hydrogen peroxide can then be electrochemically detected, but this requires a high electrical potential to achieve good selectivity [10]. To overcome this, second generation glucose sensors use electron acceptors, such as ferrocene, to mediate the oxidation of glucose and transfer electrons to a working electrode [11]. As hydrogen peroxide is no longer the detection molecule, glucose-1-dehydrogenase (GDH) can be used as the glucose oxidizing enzyme. Third generation glucose sensors use direct electron transfer between the oxidizing enzyme, such as GDH-PQQ (pyrrolo quinolone quinone), and the electrode using electron conductive hydrogels [5].

To calculate the concentration of glucose most commercially available devices use amperometry, where the current is measured at the working electrode while the electrode is held at a constant voltage [12]. After an initial period, the current measured is proportional to the rate of glucose oxidation, which is diffusion limited. As Fick’s first law states, the diffusion rate is proportional to the concentration of glucose, making the current proportional to the glucose concentration [13].

The main type of device used to perform electrochemistry is a potentiostat [14]. The basic requirement of a potentiostat is to control the voltage between two electrodes while measuring the current passing between them, this is a 2-electrode configuration for a potentiostat. To make accurate measurements, the electrodes have to pass a current while maintaining a standard / reference voltage between them. Because a current passing through an electrode causes a voltage change that interferes with keeping the voltage between the electrodes constant, a third electrode is often used for a 3-electrode configuration. In the 3-electrode configuration, the chemical reactions occur on the working electrode surface while the reference electrode provides a stable reference potential, and not should not pass current, and a counter (or sometimes called the auxiliary) electrode provides enough current to the solution to keep the potential between the reference and working electrode at the desired voltage [15].

As the three electrodes have different roles, different materials can be used for each electrode in the 3-electrode configuration. The working electrode is selected based on the chemical reaction to be studies [16]. The counter electrode is selected to have a large conductivity to supply current and to be stable and not degrade or oxidize during the experiment, while still passing current [17]. For this reason, noble metals are often used, with platinum being especially prominent. As the reference electrode needs to provide a reference voltage, it needs to have a well-known electrode potential, i.e. a known electrical potential between the electrode material and the electrolytes in solution. Common reference electrodes include the saturated calomel electrode and silver / silver-chloride which are stable and have well characterized electrode potentials.

There have been many potentiostat designs reported recently with many designed with discrete integrated circuits (ICs) that are connected with a printed circuit board (PCB) [18–20]. Many of these designs have shown very good performance [21], small size [22–24] and low cost [25–27]. Most open source potentiostats cover the voltage ranges of ± 1.5 V, and voltages in excess of this results in electrolysis of water, making larger voltages less useful in aqueous solutions. And most of the potentiostats are limited to around 0.1–0.2 mA of current as they have mostly been designed for analytical chemistry purposes, though the DStat does have the capability to measure up to 10 mA of current, see Table 1 for a comparison of previous works [21]. Almost all implement the most common electrochemical techniques of cyclic voltammetry, amperometry, and potentiometry, while the DStat, uMed and UWED also implement square-wave voltammetry and differential pulse voltammetry [19,21,28]. The uMed and UWED are also notable for their incorporation of a wireless interface into their open source design making them a good fit for remote healthcare applications.

**Table 1:** Comparison of different potentiostats.

Potentiostat	Voltage range	Current range (mA)	Noise	ADC resolution	Cost	Max sampling rate (kHz)	DAC resolution (mV)
PSoC-Stat	± 2 V	0.1^a^	26–2.5 nA^b^	12-bits^c^	$10	50	1
uMed	± 2 V	0.156	5–0.5 nA^d^	16-bits	~$25^e^	0.8	0.05
DStat	± 1.5 V	10	10 nA—0.2 pA^f^	24-bits	~$120	30	0.046
CheapStat	± 0.99 V	0.05	not listed	12-bits	$80-$135^g^	2	1

^a^ This range can be extended using an external resistor, a 10 mA range is possible with a 100 Ohm resistor between P[0][4] and P[0][7]

^b^ Noise is dependent on the current range used and is explored in S5 Supporting Information

^c^ ADC can be configured to have a higher resolution if desired

^d^ Paper lists a circuit resolution of 5 nA and that they average 10 measurements to get a 0.5 nA noise limit

^e^ Paper lists parts as costing $25 if parts for 1000 units are ordered, but cost does not count assembly

^f^ Noise is dependent on current range used

^g^ The paper states $80 to build from parts, a single assembled unit can be purchased for $135 from iorodeo.com

However previously reported potentiostats require the user to manufacture a PCB and to solder the electronic components onto the board. As these potentiostats are designed with small, surface mounted ICs, they require more skilled technicians and sophisticated tools to solder together, as compared to through-hole connections. This creates impediments to chemists and other scientists who want to use a potentiostat. There are also many reports of single chip potentiostats manufactured with complementary metal-oxide-semiconductor (CMOS) and related technologies [29–31]. These require even more specialized skills and equipment than the PCB / IC based potentiostats. This paper describes a potentiostat that can be made with commercially available parts without making a PCB or any soldering. To do this we use a Programmable System on a Chip (PSoC), a single IC microcontroller that includes with the CPU core, an array of other digital and analog electronic components. The parts needed are 1.) PSoC 5LP board (such as a CY8CKIT-059, CY8CKIT-050, or FreeSoC2; these are PCBs made with a PSoC 5LP attached that are made available to develop applications on, i.e. development boards), 2.) computer, and 3.) some electrical connectors. To attach the electrical connectors to the board, a conductive glue can be used, or they can be soldered on; which should be easy for most as the pins are through-hole and are easier to attach than surface mounted electrical components.

There are some tradeoffs for the convenience of using a single chip potentiostat such as, the Opamps cannot be selected to optimize noise or the offset voltage. For example the Opamps used in the DStat were selected for this criteria and have a noise of $6.5\;nV/\sqrt{Hz}$ and an offset of < 0.2 mV, in comparison the Opamps of the PSoC 5LP have a noise of $45\;nV/\sqrt{Hz}$ and an offset of up to 12 mV (for the reference and working electrodes combined). Also the inputs of the PSoC 5LP are programmable general purpose input-output pins which cause them have a leakage current of up to 2 nA while the DStat Opamps, with their dedicated inputs designed for low currents, only have an input bias current of 200 fA.

The ability to develop a potentiostat on a single chip is due to the power of the PSoC by Cypress Semiconductor, San Jose, California, which incorporates a microcontroller together with programmable analog components such as operational amplifiers (Opamps), comparators and transimpedance amplifiers. Together with the free Integrated Development Environment (IDE) PSoC Creator^™^, Cypress Semiconductor, San Jose, California, researchers with limited resources can now develop scientific and biomedical equipment quickly and with fewer components than was possible before. This can aid the growing movement towards developing open source equipment [32,33]. Just as 3D printers allows others to share the equipment they develop on the internet where others can download the designs and print them on their own 3D printer; the PSoC allows electronic designs, with both microcontroller and circuit elements, to be shared over the internet where others can use them by programming them into a PSoC device [34].

This paper shows how to produce a potentiostat using a single PSoC 5LP. To demonstrate our device, a low cost (~$10) CY8CKIT-059 was used, which can be ordered from any electronics distributor. In order to access the device, we showed that it could quantify the amount of vitamin C in orange juice, measure glucose levels with a glucose strip, and detect and quantify the amount of lead in water.

## Material and methods

### Design

The potentiostat was designed with PSoC Creator 4.1, Cypress Semiconductor, San Jose, California, a free IDE for programming PSoCs. The device used a PSoC 5LP (part number CY8CKIT-059, purchased from Mouser Electronics), which has integrated analog components into a single chip with an ARM Cortex-M3 CPU.

### Voltage control circuit

**Fig 1:**
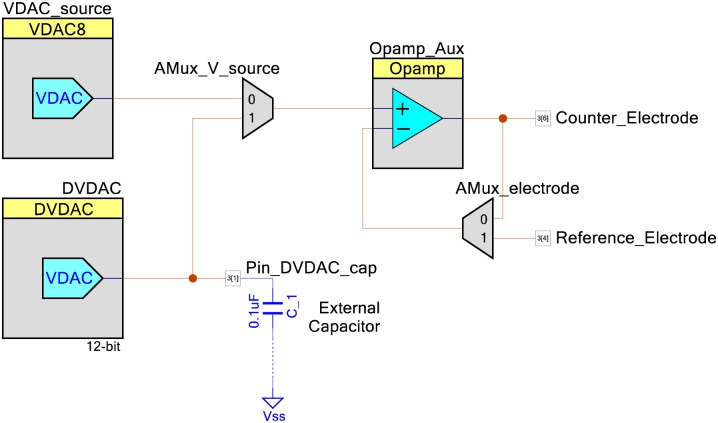
Circuit to control the reference and counter electrodes. One of two DACs can be used to control the voltage, based on if the external capacitor is installed. An Opamp buffers the DAC voltage and an analog multiplexer is used to select if 2 or 3 electrode experiments should be performed.

To control the voltage between the electrodes we use the circuit shown in Fig 1. The device can use an 8-bit voltage digital to analog converter (VDAC) or a 12-bit Dithering VDAC (DVDAC). The DVDAC is comprised of an 8-bit VDAC where the voltage of the DAC is quickly switched between 2 values. This makes the output the weighted average of the values written, which can be used to increase the resolution of the DAC. This switching causes noise on the output of the DVDAC so that a small capacitor (100 nF) has to be placed on its output to smooth out the voltage. Depending if the user has installed the external capacitor or not, they can choose what DAC to use to drive the common electrode by using an analog multiplexer (AMux) to select the DAC. The user interface asks the user if the DVDAC capacitor was installed and programs the device accordingly. The user’s choice is then saved in the electrically erasable programmable read-only memory (EEPROM). An operational amplifier (Opamp) is used to buffer the voltage and to provide feedback from the reference electrode [35,36]. The build in Opamp has a bandwidth of 3 MHz [37]. The device can be operated in the standard 3 electrode mode or in a 2 electrode mode by setting the appropriate channel on the electrode AMux, which can be done through the user interface. This circuit will pass current through the common electrode pin until the voltage on the reference electrode pin is the same voltage as the DAC. The DAC’s voltage is set by the firmware depending on the electrochemical parameters inputted into the device.

### Current measuring circuit

To measure the current, we use a current to voltage converting circuit, a transimpedance amplifier (TIA). The analog voltage from the TIA is then converted to a digital signal using an analog to digital convert (ADC).

**Fig 2:**
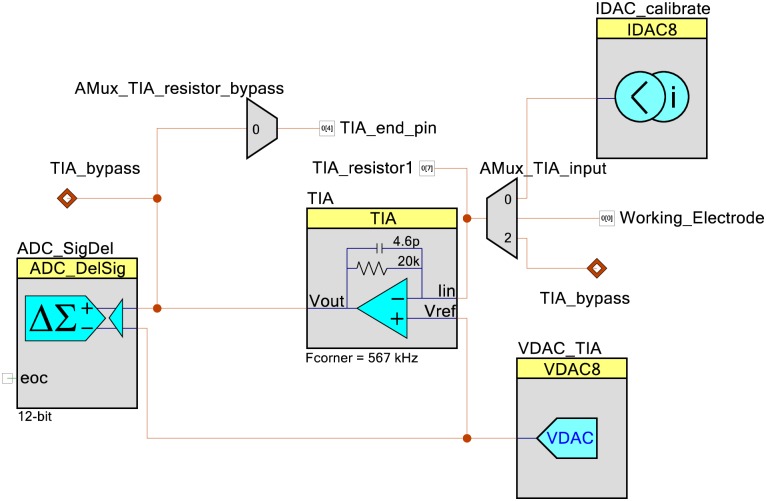
Current measuring circuit. A transimpedance amplifier (TIA) and a DAC are used to set the voltage and measure the current passing through the working electrode. The TIA output voltage is passed into a delta sigma ADC, which converts the analog signal into a digital signal. A current DAC is used to calibrate the TIA / ADC signal chain. If an increase in the maximum current is needed, pins are made available that external resistors can be connected to.

Fig 2 shows the circuit used to set the working electrode voltage and to measure the current passing between the common and working electrodes. To allow the device to work from a single power supply while still performing electrochemical experiments at negative potentials, a virtual ground is used. An 8-bit VDAC is used to make a virtual ground at 2.048 V so that the DAC driving the common electrode can make a -2.0 to +2.0 Volts difference between the common and working electrodes, which is a wider range than is needed for most electrochemical experiments.

The current that passes through the working electrode is fed into a TIA, with a -3 db cutoff frequency of 567 kHz [38]. As the input impedance of the Opamp is high, the current goes through the resistor of the TIA. This causes a voltage that is the product of the current and the resistance according to Ohm’s law (V = I*R). This voltage is then measured by a delta sigma ADC. The ADC is in differential mode, where the voltage is calculated from the difference of the TIA output voltage and the virtual ground voltage. The ADC values are then sent to a computer where they are used to calculate the current that was passed between the counter and working electrodes.

The TIA of the PSoC has a variable impedance that can be controlled by the firmware. The impedance can be one of 8 levels between 20 kilohms and 1 megaohm. To do this the TIA uses switch capacitors and because of how the switch capacitors are manufactured, there can be a large variability in the actual impedance of the TIA (the datasheet says between -25 to +35% of the indicated value). To correct for this the current measuring circuit has a self-calibrating routine. An 8-bit current DAC (IDAC) is used to passed 5 different current levels into the TIA / ADC circuit. The resulting ADC values are then used to calibrate the TIA / ADC signal chain by using a linear interpretation of the calibration data to create the ADC counts to voltage conversion factor and to adjust any voltage offsets. To control when the calibration current or the working electrode current is passed into the TIA component an AMux is used. As the smallest impedance of the TIA is 20 kilohms, the device is limited to 100 μA of current. To give the user the option of increasing the current range, 2 analog pins (P[0][7] and P[0][4]) are made available that the user can place a resistor between with an AMux used to select when the external resistor should be used.

S1 Supporting Information contains the analog circuit diagram drawn with an external schematic program using more conventional symbols and the different analog multiplexer connection options shown as typical switches for an additional reference guide to the analog circuitry.

### Peripherals

**Fig 3:**
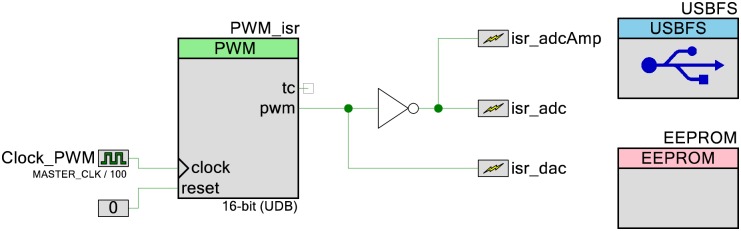
Peripheral components. To set the timing of when to change the DAC controlling the electrode voltage and when to measure the current, a PWM is used to trigger a set of interrupt service routines (isr). Communication to and from the device is done through a USB component, and an EEPROM is used to save what DAC the user wants to use.

Fig 3 shows the other peripherals used by the device. To control the timing of when the DAC voltage should be changed and the ADC values measured, a pulse width modulator (PWM) is used to trigger interrupt service routines (isr). The period and counter values of the PWM are controllable by the user to set different sampling rates. An EEPROM is used to save what DAC the user wants to use. To allow the user to configure the device properly and to export the data from the device to a computer, a Full Speed USB component is used. The USB uses 2 endpoints, an interrupt OUT endpoint that polls the computer ever 10 ms that the user can use to send instructions to the device and a bulk IN that polls the computer every 1 ms that sends the data to the computer to display to the user.

### Firmware development

The firmware for the device is located at github.com/KyleLopin/PSoC-Potentiostat. The firmware was developed in PSoC Creator 4.1 developed by Cypress Semiconductor. Figs 1–3 are all made in the TopDesign window of PSoC Creator, which will configure the analog portion of the PSoC 5LP chip.

### Graphical user interface

**Fig 4:**
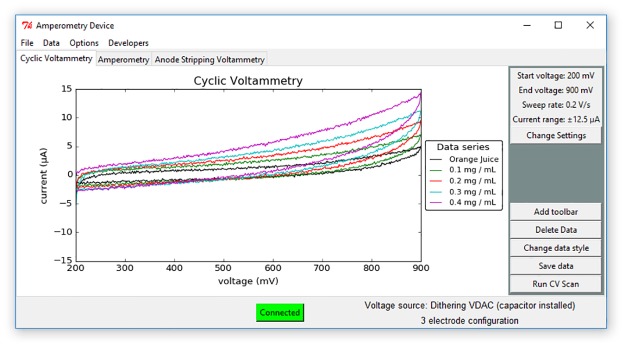
Screenshot of GUI to control the potentiostat. Different electrochemical techniques use different tabs in the main GUI. Important information for each experiment type is displayed to the user and a pop-up interface allows the user to change each parameter. The voltage source and electrode configuration settings are displayed to the user.

A screen shot of the graphical user interface (GUI) is shown in Fig 4. The GUI is written in the python software language, version 2.7. The source code can be found at https://github.com/KyleLopin/Potentiostat_GUI and a copy of the source code has been compiled into an executable and can be found at https://github.com/KyleLopin/Potentiostat_GUI/releases.

The user can select different electrochemical techniques by selecting the appropriate notebook. The data is displayed using the matplotlib library. USB communication is done using pyUSB and the libusb-win32 libraries. All libraries and the USB backends have been compiled into the single executable file.

### How to create the device

**Fig 5:**
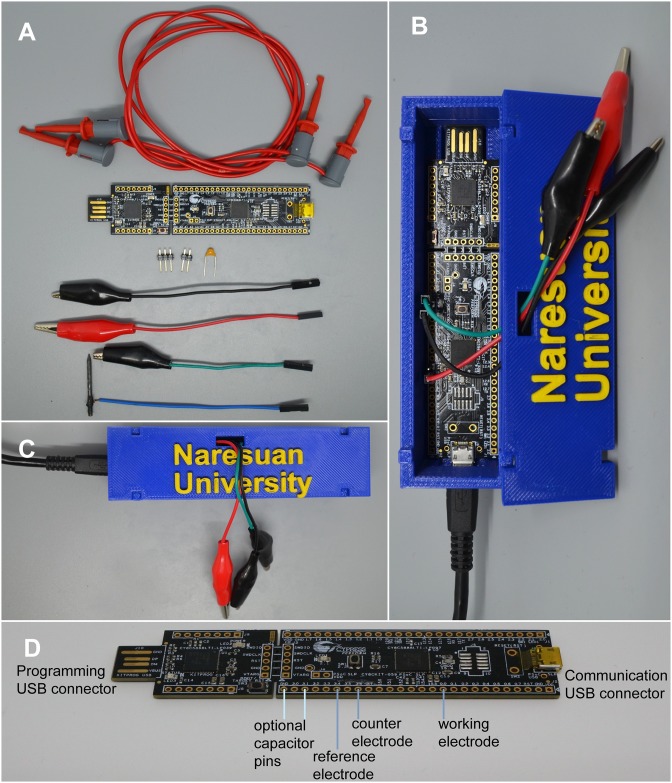
Potentiostat parts and completed device. A) Photo of all the components needed to develop a single chip potentiostat and perform the experiments in this paper. *From the top*: EZ-Hook electrical connectors used to connect the device to glucose strips; CY8CKIT-059 that has the PSoC 5LP that contains all the electrical components needed for a potentiostat; headers that can be connected to the board, with either solder or conductive glue (Conductive glue is not as strong over time but requires less equipment and skill to use); The DVDAC capacitor is optional but will increase the resolution of the potentiostat; 3 alligator clips with female jumper cables ends to attach electrodes to the device; a pencil lead electrode attached to a jumper wire with electrical glue. B) Photo of the assembled device with a case. C) Device assembled in its case. D) Close up of the CY8CKIT-059 with labels for where the electrodes, capacitor and USB connections should be attached.

The following steps allow anyone to create the device shown in this paper. All components used for the experiments in this paper are shown in Fig 5A, and a completed device is shown in Fig 5B and 5C. Fig 5D shows a close up of the CY8CKIT-059 with the pins labeled. A video of how to create this device is available at https://youtu.be/OCPO-TYS1mw.

1. Obtain a CY8CKIT-059 and plug the USB programmer end (the male end) into a computer. Note: The board has exposed electrical components, so an insulating enclosure for the board is beneficial.
2. Download the free program PSoC Programmer from Cypress Semiconductor found at http://www.cypress.com/products/psoc-programming-solutions
3. Load the Potentiostat.hex file located at https://github.com/KyleLopin/PSoC-Potentiostat, or S1 File, into PSoC Programmer.
4. Attach wires to pins 0.0 (working electrode), 3.4 (reference electrode), and 3.6 (counter electrode) using either conductive glue, solder or alligator clips.
5. Use a USB cable to connect to the female end of the CY8CKIT-059.
6. Download the free program Zadig, at http://zadig.akeo.ie/, to install the windows UBS drivers.
7. Select “List all devices” from the options menu in Zadig, select the “Naresuan Potentiostat” device, select the libusb-win32 driver and click on the Install Driver button.
8. Download and run the executable file NU.Potentiostat.exe at https://github.com/KyleLopin/Potentiostat_GUI/releases, or unzip it from S2 File.
9. Optional: A small capacitor (100 nF) can be connected between pins 3.1 and ground on the board so that the more accurate dither VDAC can be used.

### Experimental setup

#### Ferricyanide experiment

To compare the performance of our device to a commercial potentiostat, we performed cyclic voltammetry on 5 mM Potassium ferricyanide (K_3_[Fe(CN)_6_]) (Lab Scan, Gliwice, Poland) in 1 M KCl (Sigma Aldrich) with our PSoC-Stat and an EmStat3 potentiostat (PalmSens BV, Utrecht Netherlands). We used a conventional three-electrode electrochemical cell with 3 standard electrodes. We used a glassy carbon electrode (BASi^®^, Indiana, USA) for the working electrode, a platinum electrode (Nilaco, Tokyo, Japan) for the counter electrode, and a standard Ag/AgCl electrode (BASi^®^, Indiana, USA) was used as the reference electrode. The experiments were run from 1000 mV to -400 mV with a scan rate of 10 mV/s.

#### Ascorbate determination in orange juice

To determine the amount of ascorbate (Vitamin C) in orange juice, we used the technique of standard additions. We used a graphite electrode, a 20 mm thick 2B pencil ‘lead’ (brand Rotring, purchased from a local book store with coordinates: 16°44’43”N 100°11’36.2”E), for the working electrode and 99.9% pure Ag wire (purchased from a local jewelry store with coordinates: 16°48’45.8”N 100°15’43.6”E) for the counter and reference electrodes. The Ag wires were put in a container with bleach for 5–15 minutes, until they were observed to turn from silver to black, to coat the outside of the wire with a Ag/AgCl layer. The wire was then rinsed with deionize water to remove any bleach residue before use. The counter and reference electrodes were connected using alligator clips. Conductive glue was used to connect a wire to the working electrode. A picture of the electrode attachments is shown in S2 Supporting Information and the STL file to 3D print the electrode holder is in S4 File. We tested platinum and gold wires for the counter electrode and got similar results, but we chose to show the results from the cheapest set of electrodes we tested. To limit the current and to keep the surface area of the working electrode constant between experiments, even if the fluid level was slightly different, we coated the working electrode with dental wax and used a flame to expose just the tip of the electrode. 0.5 mm pencil ‘lead’ was also tested but was found to be difficult to use because they would often break between experiments. Type 2H pencil ‘lead’ was also used but had very poor results with much smaller currents than 2B.

We mixed 1 part orange juice, 1 part 3 M KCl (to increase the conductance) and 1 part of added ascorbate to make our standard addition solutions. The orange juice was purchased from a local grocery store (coordinates: 16°48’45.8”N 100°15’43.6”E) and was made of Si Thong oranges. The KCl used in the 3M stock solution was purchased from Merck (CAS-No: 7447-40-7) and Sodium L-ascorbate was purchased from Sigma Aldrich (A4034-100G). We then gave a cyclic voltammetry sweep from +200 mV to +900 mV with a sweep rate of 0.1 V / second. A rolling mean of 2 samples was used to eliminate the 50 Hz noise picked up from the power lines in the building. Because a sweep rate of 0.1 V/second samples at 100 Hz, this rate picks up 50 Hz as a very high frequency noise in the signal. This noise could be eliminated by removing or shortening the lead wires connecting the device to the electrodes, but not by running the experiments on a battery powered laptop. This suggests the noise is picked up from electromagnetic interference.

To calculate the value of ascorbate in our sample of orange juice we measured the current level at +550 mV, as was used in [25,39], for all samples. Using potentials higher than this can lead to species other than ascorbate oxidizing [39]. Next the current versus the added ascorbate was plotted [25]. By interpreting a line through the samples back to a zero current (the x-axis intercept) we can calculate the amount of ascorbate in the orange juice sample. The error of the standard additions was calculated using the following equation:

$$s_{x}=\frac{s_{y}}{\left|m\right|}\sqrt{\frac{1}{n}+\frac{{\overset{-}{y}}^{2}}{m^{2}{\sum (x_{i}-\overset{-}{x})}^{2}}}$$

where s_y_ is the standard deviation of the residuals given by the following equation:

$$s_{y}=\sqrt{\frac{\sum {\left(y_{i}-m∙x_{i}-b\right)}^{2}}{n-2}}$$

and *m* is the slope of a line fitted to the data, *n* is the number of standards measured, $\overset{-}{y}$ is the average current measured, *x*_*i*_ is the concentration of the added standard, $\overset{-}{x}$ is the average of the standards added, y_i_ is the current measured, and b is the calculated y-intercept [40].

#### Glucose measurements

To measure glucose, we purchased Accu-check Performa glucose test strips, Roche Diabetes Care, Inc., Indiana, USA. We used 2 mini-hook test clips to connect the outside electrodes of the glucose strips to the device to show a complex connector was not needed to use commercial strips, see S3 Supporting Information.

The glucose samples were prepared by making a 2000 mg/dl glucose (Ajax Finechem: 783-500G) stock solution with 1X phosphate buffer solution (PBS) (Gibco: 14190–144) and then mixing the appropriate amount of the glucose stock with 1X PBS to make our testing samples. An amperometric experiment was performed for 6 seconds with the working electrode set at +500 mV [41], to receive electrons from the glucose oxidizing enzyme, GDH-PQQ [42], and the current was recorded at a rate of 1 kHz [43]. Initial tests showed that the readings were noisy due to power line noise so a 50 points rolling mean was used to smooth out the signal. To quantify the amount of glucose we took the average current from 4.8 to 5 seconds.

#### Lead determination in water

To measure the amount of lead in water we first made a 2 M sodium acetate (Sigma Aldrich: 53272-250G) stock solution, an 800 mg/L stock solution of bismuth nitrate (Sigma Aldrich: 383074-100G) and a 200 ppm lead citrate (from Electron Microscopy Science: CAS-No: 512-26-5) stock solution. Next we used deionized water and made samples with a final concentration of 0.1 M sodium acetate, 4 mg/L of bismuth and the desired amount of lead for each sample.

We used a 20 ml glass vial as our chemical chamber with a gold wire as the working electrode and a Ag/AgCl wire as the counter and reference electrodes. The chamber was placed on top of a stir plate and a Teflon stirrer was used to stir the solution during the plating step, see S4 Supporting Information for a picture of the setup.

For the anodic stripping voltammetry experiments we used a cleaning step of +500 mV for 30 seconds, a plating step of -1100 mV for 5 minutes with the solution being stirred for the first 4.5 minutes. A linear sweep from -1100 mV to +500 mV at a rate of 0.1 V/second was given after the plating step to strip the lead. Between readings the electrode is electrochemically cleaned by applying a +500 mV potential for 30 seconds while the solution is stirred to remove any lead or bismuth from binding to the electrode between experiments [44].

Additional experiments were also performed with a plating time of 10 minutes (included in the S1 Dataset) but they gave less clear results than the 5 minute plating time experiments. Also a carbon electrode was tested but the background current was so large that it was more difficult to measure the stripping current. While others have shown more sensitivity with carbon electrodes, they usually require a special coating, such as Nafion, to reduce the capacitive current [45,46]. We chose to use a simpler working electrode composed of an uncoated 1 mm diameter, 99.7% pure gold wire that was purchased from a local jewelry store (coordinates: 16°48’45.8”N 100°15’43.6”E).

## Results and discussion

### Device characterization

**Fig 6:**
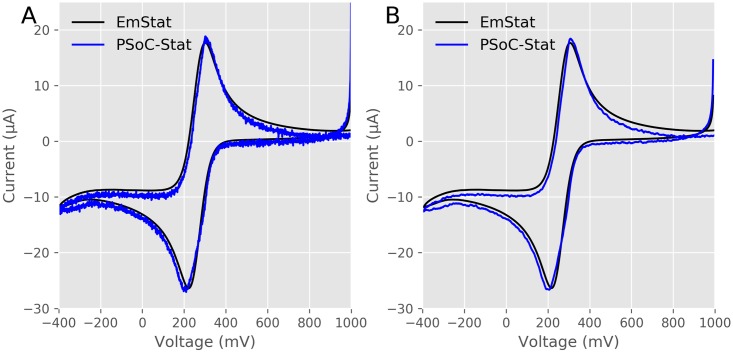
Comparison to a commercial potentiostat using standard electrodes. A) Raw current traces of cyclic voltammetry experiments in 5 mM ferricyanide using an EmStat (black) and PSoC-Stat (blue) with the voltage in reference to a Ag /AgCl reference electrode. B) Currents from A with the noise removed using a 10 point rolling sample.

To verify our device, we compared it to a commercial potentiostat, an EmStat from PalmSens, using standard electrodes. Fig 6 shows the results. Fig 6A shows our data in blue and a commercial potentiostat in black, performing cyclic voltammetry on a 5 mM ferricyanide solution in 1 M KCl with a scan rate of 10 mV/second. Both show the characteristic reversible oxidation and reduction peaks. There are slight differences between the traces, some of which could be due to electrochemical variations and temporal changes in the electrodes [19,21]. The PSoC-Stat can also have a slight voltage shift from the onboard Opamps. For potentiostats built from discrete ICs, the Opamps can be selected that are specialized for their applications. For potentiostats, the Opamps should be chosen to have no voltage offset. For the PSoC-Stat, we had to use the Opamps in the PSoC 5LP which can have a voltage offset. The Opamp that drives the counter electrode can have a maximum of 2 mV offset (though it will typically have 0 offset [37]) and the Opamp in the TIA circuit can have a maximum of 10 mV offset (though it typically will have 0 offset [38]). There are some tradeoffs for using a fixed single chip to build a circuit, and if an electrochemical experiments needs to be performed with an absolute accuracy of greater than 12 mV, the PSoC-Stat cannot meet that need and a more accurate potentiostat should be invested in.

Also the current is noisier for the PSoC-Stat, most likely due to the TIA using switched capacitors to emulate the variable resistor used to change the current range, which will introduce a high frequency noise. In Fig 6B we show the data from Fig 6A with a 10 point rolling mean which removes most of the noise from the data. This is the strategy used by the uMED potentiostat [19]. The ADC of the PSoC-Stat has a sampling rate of 50 kHz, a future strategy to have the hardware remove the noise would be to collect multiple samples per data point and average them (even at 1 V/second the ADC can collect over 10 samples per step). Also the PSoC 5LP has a digital filter blocks (DFB) that can be configured to filter out the noise. Or post data acquisition filtering can be performed as was done here. The GUI has an option in the menu to implement a rolling mean with a variable number of samples.

A more detailed characterization of the circuit noise is given in S5 Supporting Information and Table 1 is given to compare our noise and other important parameters to the other open source potentiostats developed.

### Orange juice standard additions

**Fig 7:**
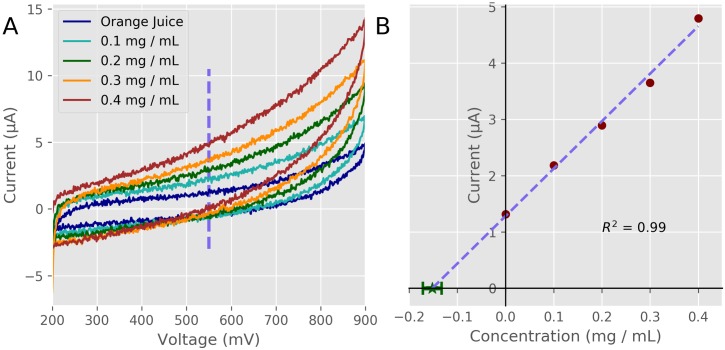
Cyclic voltammetry experiments. A) Raw current traces of cyclic voltammetry experiments in orange juice with different levels of added ascorbate. Voltage is in reference to a Ag /AgCl reference electrode. B) Standard addition results of the cyclic voltammetry experiments. The current value at +550 mV was used and a fitted line to the data was used to calculate the amount of ascorbate in the orange juice.

To demonstrate the capabilities of our device we performed standard addition experiments to quantify ascorbate (Vitamin C) in orange juice using cyclic voltammetry as shown in Fig 7. This experiment is exceptionally well positioned to be an introductory analytical chemistry lab as all the materials are inexpensive, non-hazardous and demonstrates the technique of cyclic voltammetry and standard additions [26,39,47].

Ascorbate is redox active and will readily lose electrons (oxidizes) when the voltage of the working electrode increases above +100 mV (data not shown). The flow of electrons released from the oxidation of ascorbate is collected by the working electrode and measured by the potentiostat allowing the quantification of the oxidation rate, which is correlated with the concentration of ascorbate in the solution. By adding known quantities of ascorbate to the orange juice and measuring the increase in current, the ratio of current increase to concentration can be calculated. Taking the relationship between current and concentration with the current measured in the pure orange juice sample, the amount of ascorbate in the orange juice can be determined. Because the ratio of current to concentration is the slope of the fitted line, by extending the fitted line until the current is zero, the x-axis intercept is the concentration of ascorbate in the original sample [39]. Our experiments give a result of 0.152 ± 0.019 mg/ml. This is within 2% of the value given on nutrition panel of the orange juice of 0.15 mg/ml.

### Glucose measurements

**Fig 8:**
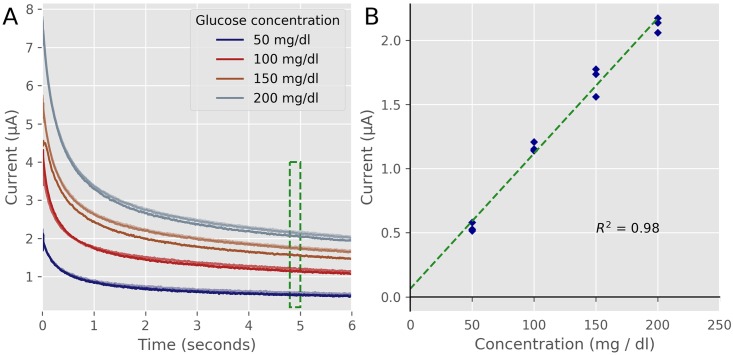
Amperometric experiments. A) Current traces measure at +500 mV using Accu-Check Performa glucose strips. 3 strips were used for each glucose sample. B) Average current between 4.8 and 5 seconds of the amperometric currents is plotted versus the glucose in the samples. Our device measured a linear relationship (R^2^ = 0.98) over the physiological range of glucose within the range of the FDA’s guidelines.

The most common electrochemical medical device in use today is the glucose meter used by diabetics to monitor their blood glucose levels [48]. To measure glucose the working electrode is coated with the enzyme glucose oxidase, that converts glucose to gluconic acid with the production of hydrogen peroxide [49,50]. The hydrogen peroxide is then oxidized on the surface of the working electrode [51]. Glucose meters use the technique of amperometry, where a constant voltage is applied between a working electrode and counter electrode while the current is measured versus time. Because the glucose strips have 2 electrodes, the 2 electrodes configuration was used for the glucose measuring experiments. The user can select the 2-electrode option from the option menu of the GUI. Fig 8 shows the results of our experiments. Fig 8A is the raw current traces measured. To calculate the amount of glucose in solution, the current traces are measure for 5 seconds and the average of the current from 4.8 to 5 seconds is plotted versus the glucose concentration, see Fig 8B [52]. Our device showed a relationship of a 10.5 nA current for each mg/dl increase of glucose in solution with an R^2^ of 0.98. At the current range used for the measurement of ±8 μA, the PSoC-Stat has resolution of 0.4 mg/dl. At this level most variation in the glucose measurements will be due to differences between glucose strips, and the temperature and humidity changes between measurements [53]. The PSoC-Stat measurements were well within the 15% accuracy needed for a glucose meter to be approved by the FDA [54].

### Lead measurements

Lead contamination has become a major problem worldwide, from the use of lead pipes and lead solder used to join copper pipes together in the water system of America [55,56], to area contamination from mining activity [57]. Because there is almost no government testing of household water, it would be beneficial for people to have an easy way to test if their water is safe for human consumption [58–61]. To measure lead in water we performed anodic stripping voltammetry (ASV) on water samples prepared with added bismuth [62]. ASV uses a negative voltage on the working electrode to “plate” (reduce) dissolved metal ions onto the electrode. To increase the sensitivity of the electrode, bismuth was added to the solution [63]. During the plating potential Bi^2+^ will plate onto the electrode with Pb^2+^ and form a “fused alloys” [64]. The electrode’s voltage is then ramped up to “strip” (oxidized) the ions off. By measuring the currents from oxidation as a function of voltage, the amount of metal ions in solution can be quantified.

**Fig 9:**
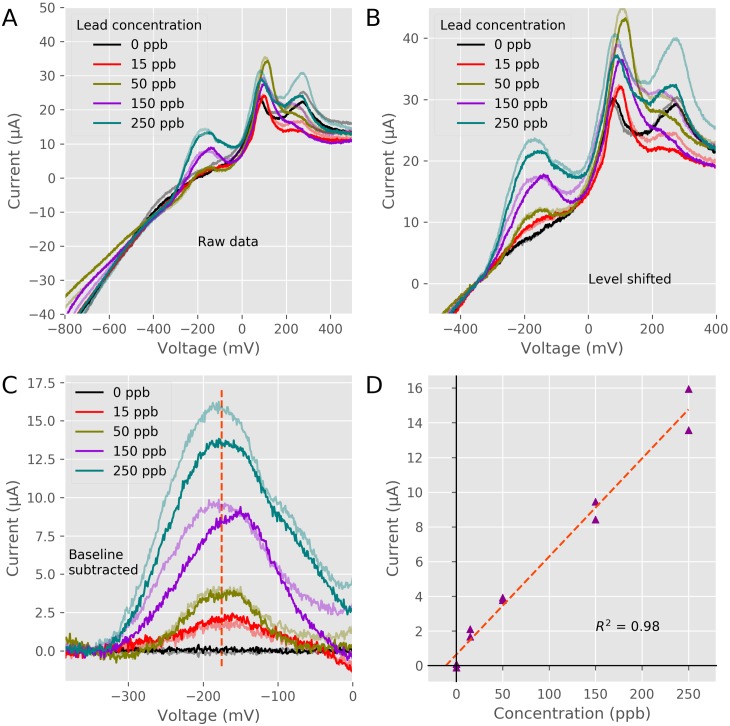
Anodic Stripping Electrode experiments. A) Raw current traces of the stripping step with added Pb^2+^. Voltage is in reference to a Ag /AgCl reference. electrode. B) Raw traces normalized to the -350 mV current level. C) Current with the average baseline subtracted from all traces. D) Relationship of the current at -175 mV of the baseline subtracted traces versus the Pb^2+^ concentration.

Fig 9A shows the raw traces measured during the linear sweep of the ASV measurement. In these traces 3 current maximums can clearly be seen. The curves in the current around -200 mV occur as Pb atoms are oxidized off of the electrode to become Pb^2+^ ions, while the increased current at +100 mV and +250 mV, is the oxidation of Bismuth atoms [65]. Because there is a background capacitive current that is sensitive to the area of the working electrode exposed to solution, a small change in the height of the testing solution can affect the background current. For the cyclic voltammetry measurements in orange juice we coated our electrodes in dental wax to fix the surface area of the electrode for each experiment. This was not possible in these experiments as the bismuth and lead will bind to the dental wax and not on the electrode. To fix the background current level of our experiments we normalized each current at -350 mV, as shown in Fig 9B. To further enhance the signal from the water samples the baseline (zero added lead) samples were averaged and that averaged baseline was subtracted from all recordings as shown in Fig 9C. Taking the current of the processed signals at -175 mV showed a linear relationship between the current and the lead concentration, see Fig 9D. These experiments shows that the PSoC-Stat could measure lead at levels above the concentration approved by the EPA of 15 ppb [66,67].

## Conclusions

Over the last few years there has been a movement in creating open source laboratory instruments [68]. The wide spread adoption of 3D printers has allowed labs to produce their own equipment, while sharing their designs online for others to use and modify [69]. This in conjunction with the development of open-source microcontroller platforms (e.g. Arduino), has made it easier for researchers to develop their own electronic equipment. The Programmable System on a Chip (PSoC) is another tool that can be used by the open source community to develop electronic equipment that has many benefits compared to other microcontrollers. The PSoC 5LP incorporates a microcontroller with programmable analog components. This flexibility allows electronic devices to be made without having to make a custom PCB and connecting numerous ICs. This greatly reduces the development and production cost of these devices in resource-limited environments. We demonstrated that it is possible to develop a potentiostat using just a single PSoC and demonstrated its capabilities by determining Vitamin C levels of orange juice, showed it can be used as a single chip glucose meter, and that it can determine lead contamination in water. Different electrochemical techniques are performed by changing the look up table functions in lut_function.c and additional techniques such as differential pulse voltammetry or square wave voltammetry could be programmed into the device by adding the appropriate embedded functions. We have made our device open source so that it can be used as a reference design. Others are welcome to modify and contribute code to implement other electrochemical techniques in the PSoC-Stat at the Github repository.

## Supporting information

S1 Dataset Data files used in this paper. (ZIP)

S1 File Hex file of potentiostat. This is the file to load into the PSoC 5LP to make the device tested in the paper.
(HEX)

S2 File Graphical user interface executable. The GUI used with the device in this paper.
(ZIP)

S3 File Python files used to make figures. (ZIP)

S4 File STL files for the enclosure and holders used. (ZIP)

S1 Supporting Information Circuit diagram of our device. (PDF)

S2 Supporting Information Cyclic voltammetry of orange juice setup. (PDF)

S3 Supporting Information Glucose amperometry setup. (PDF)

S4 Supporting Information Anode stripping voltammetry setup. (PDF)

S5 Supporting Information Noise analysis. (PDF)
