# Supplementary material for: PSoC-Stat: A single chip open source potentiostat based on a Programmable System on a Chip
Source: PLoS One. 2018 Jul 25;13(7):e0201353. doi: 10.1371/journal.pone.0201353 (PMC6059476; doi:10.1371/journal.pone.0201353)
Supplement: S1 Supporting Information — (PDF) [file pone.0201353.s006.pdf]

S1: Circuit Schematic

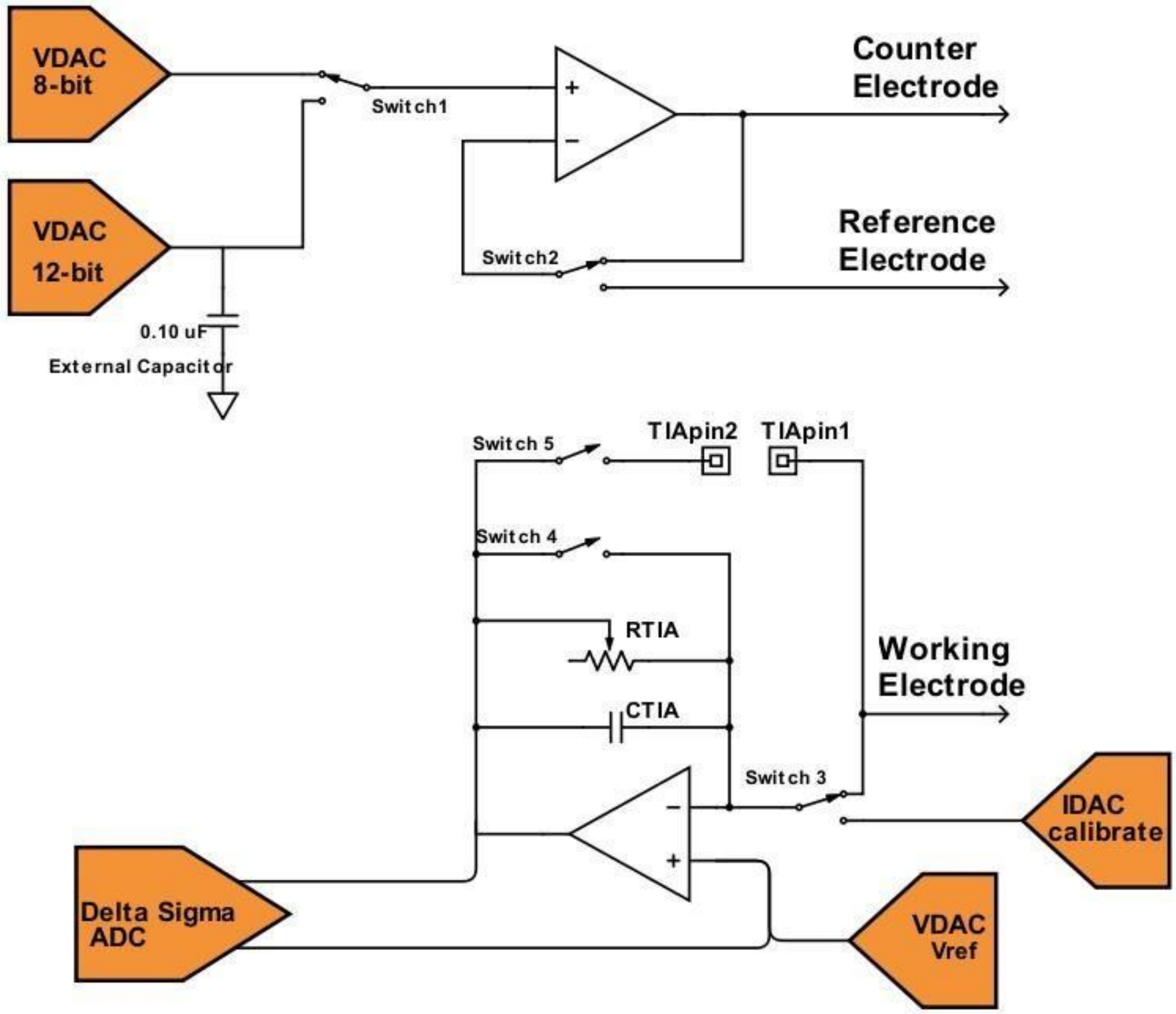

Figure A. Circuit diagram of potentiostat. Circuit diagram drawn in schemeit to show the components of the device. Switch 1 controls which DAC is used, swtich 2 controls if experiments are 2 or 3 electrode, switch 3 controls if a calibration or a measurement should be taken, switch 4 controls if the TIA should be bypassed to allow greater than 0.1 mA of current to pass (needed for the ASV plating step) and switch 5 controls if an external resistor can be used or not. An external resistor can be connected between TIApin1 and TIApin2 (P[0][4] and P[0][7])
