# Supplementary material for: PSoC-Stat: A single chip open source potentiostat based on a Programmable System on a Chip
Source: PLoS One. 2018 Jul 25;13(7):e0201353. doi: 10.1371/journal.pone.0201353 (PMC6059476; doi:10.1371/journal.pone.0201353)
Supplement: S2 Supporting Information — (PDF) [file pone.0201353.s007.pdf]

S2: Cyclic Voltammetry setup

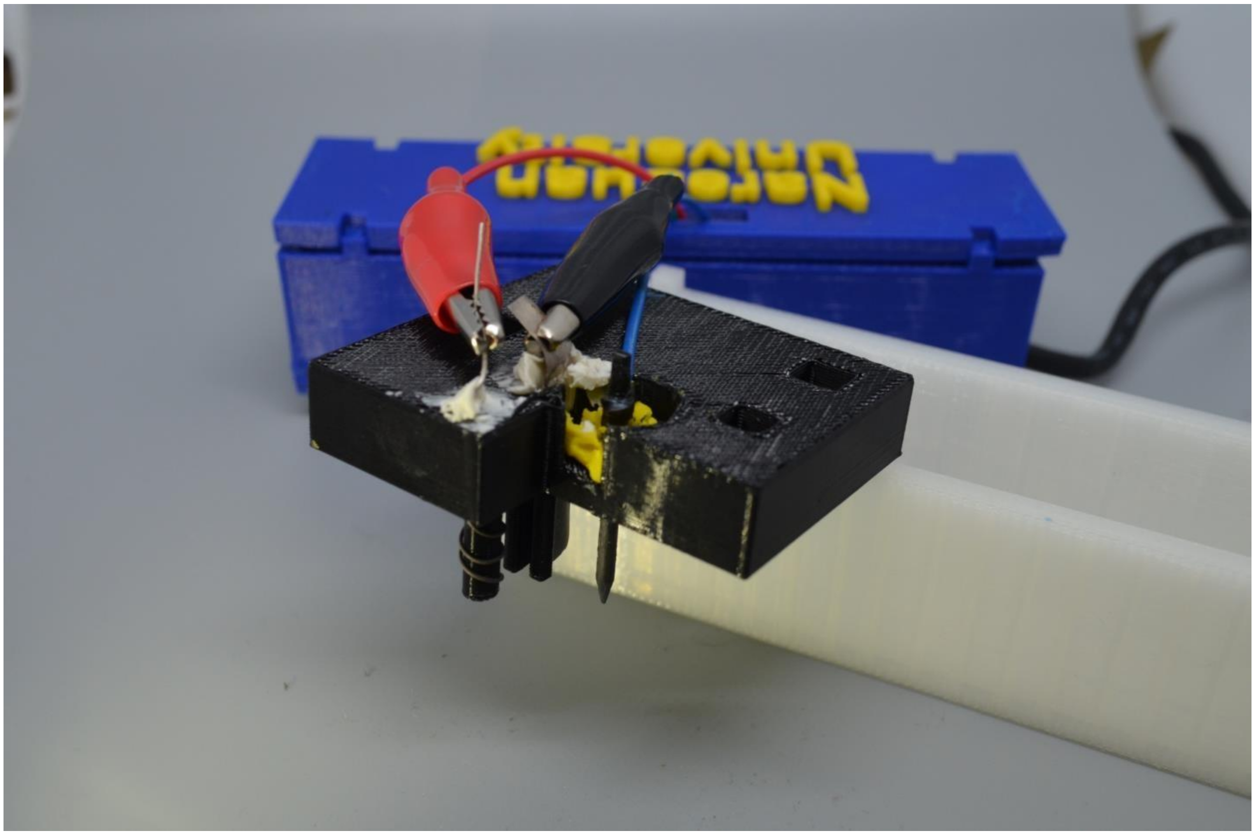

Figure A. Electrode setup for cyclic voltammetry experiment. The counter electrode is a silver/silver chloride wire wrapped around a plastic stud for support and is connected to the potentiostat with an alligator clip (red). The reference electrode is a silver/silver chloride sheet connected with an alligator clip (black). The working electrode is a 2mm diameter 2B pencil 'lead' that is connected with conductive glue. Modelling clay is used to hold the electrodes in place.
