# Supplementary material for: PSoC-Stat: A single chip open source potentiostat based on a Programmable System on a Chip
Source: PLoS One. 2018 Jul 25;13(7):e0201353. doi: 10.1371/journal.pone.0201353 (PMC6059476; doi:10.1371/journal.pone.0201353)
Supplement: S3 Supporting Information — (PDF) [file pone.0201353.s008.pdf]

S3: Glucose measurement setup

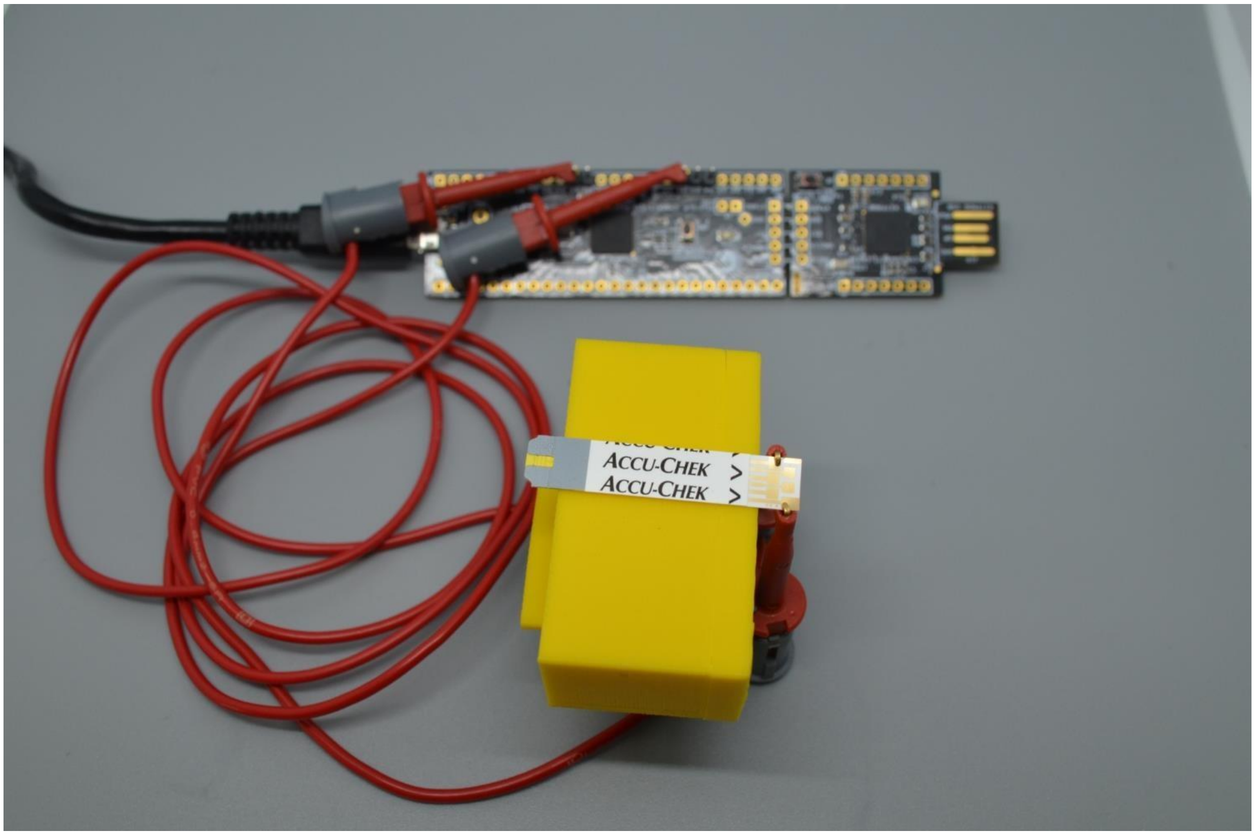

Figure A. Electrode setup for glucose measurement experiment. Hook electrodes are used to connect a glucose measurement strip to the potentiostat. The working electrode is on the left side of the test strip. Only 2 electrodes are needed for the glucose strips so the 2-electrode option is selected in the user interface.
