# Supplementary material for: PSoC-Stat: A single chip open source potentiostat based on a Programmable System on a Chip
Source: PLoS One. 2018 Jul 25;13(7):e0201353. doi: 10.1371/journal.pone.0201353 (PMC6059476; doi:10.1371/journal.pone.0201353)
Supplement: S4 Supporting Information — (PDF) [file pone.0201353.s009.pdf]

S4: Anode stripping voltammetry setup

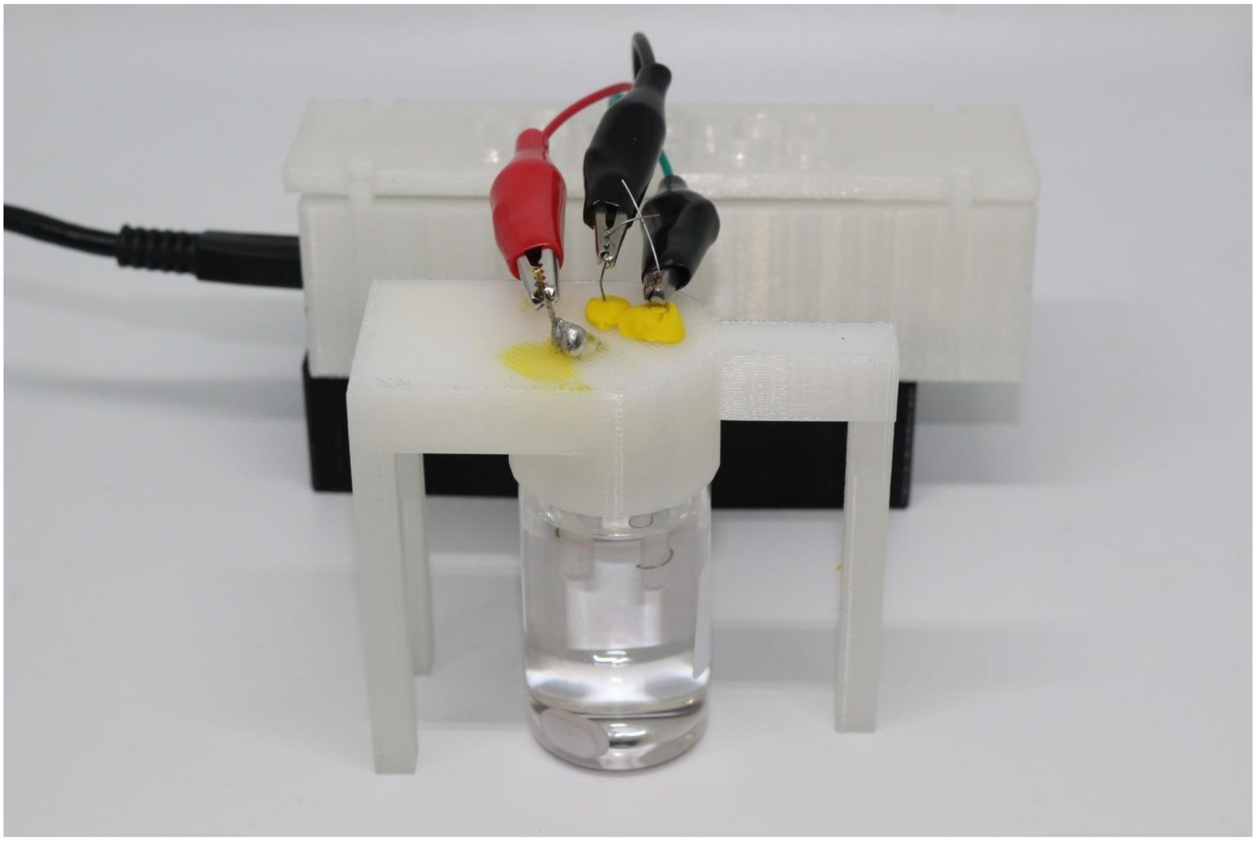

Figure A. Electrode setup for anode stripping experiment. The working electrode is a gold stud with a ball of solder on the top to keep the length inserted into vial stays the same. The working and reference electrodes are made of silver/silver chloride wires wrapped around a plastic support stud. All electrodes are connected with alligator clips.
