# Supplementary material for: PSoC-Stat: A single chip open source potentiostat based on a Programmable System on a Chip
Source: PLoS One. 2018 Jul 25;13(7):e0201353. doi: 10.1371/journal.pone.0201353 (PMC6059476; doi:10.1371/journal.pone.0201353)
Supplement: S5 Supporting Information — (PDF) [file pone.0201353.s010.pdf]

S5: Noise Analysis

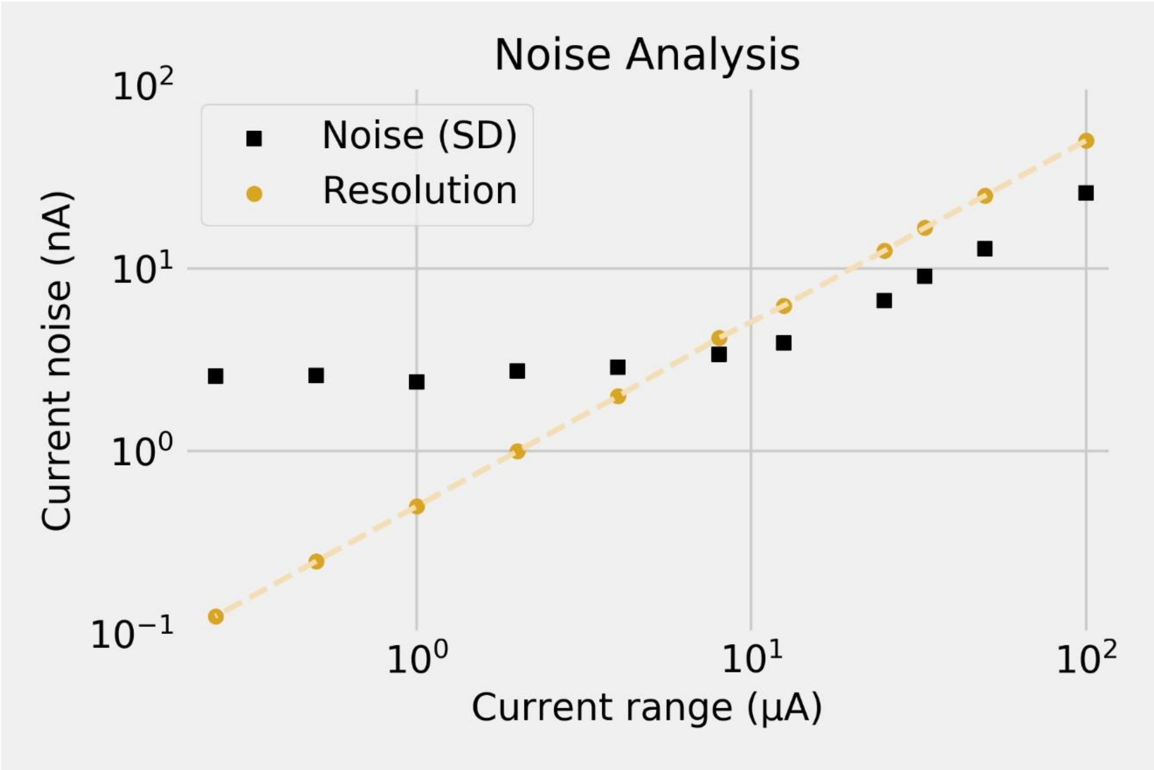

Figure A. Noise analysis of our circuit. The light colored line is the sampling resolution of our TIA / ADC circuit (in μA) and the black symbols are the experimental noise measured (in nA) without electrodes attached. For the larger current ranges, the noise of the system is below the resolution, indicating that the ADC resolution can be increased from its 12-bit setting to increase the resolution, in practice though the electrochemical noise is usually larger than the circuit noise and the resolution used. At resolutions greater than 4 μA the noise is the limiting resolution, meaning using a smaller current range is not practical as you will gain no extra resolution.
